# Surgery of the Brain and Spinal Cord

**Published:** 1903-05-16

**Authors:** 


### SURGERY OF THE BRAIN AND SPINAL CORD.

(*Concluded from page* 99.)

**Spinal-cord Tumours.** — Collins [6] contributes a long and valuable article on this subject. A previous one by the same author deals with brain tumours.[7] He says spinal-cord tumours are more susceptible to surgical treatment than brain tumours and a greater percentage recover, but, nevertheless, the majority of spinal-cord tumours prove fatal through inability to diagnose and localise them on account of their nature and extent and because of the jeopardy to life that operation brings. Spinal-cord tumour is rare in comparison with brain tumour and its diagnosis much more difficult. The general symptoms may be: sensory, comprising pain and various disturbances of normal sensation; motor, comprising spasticity, twitchings, painful cramp and paralysis, and increase of myotatic irritability; visceral, referable chiefly to the bladder and rectum; trophic, consisting of muscular atrophy, and bed-sores; and topical, comprising tenderness over the spine and sometimes deformity. The two most difficult things in the diagnosis are to determine whether the tumour is intra- or extra-dural, and at what segment of the cord it is situated. In intramedullary tumours paraplegia is usually not so complete as with tumour of the meninges, but the great point in the diagnosis between the two is the pain which in intramedullary tumour is rarely an initial symptom but is almost invariably so in tumour of the meninges, and is of great severity. Another symptom in favour of extramedullary situation is the limitation of the motor phenomena, the beginning on one side or in one extremity, or the preponderence of paresis on one side. In order to determine in what segment of the cord the tumour is located it is necessary to make out carefully the limits of anæsthesia and the muscles paralysed. In connection with the former, it must be remembered that the tumour is very often situated higher than the anæsthesia would indicate, and the consensus of opinion would place this 2 to 4 inches above the highest level of the anæsthesia, and more often the latter figure than the former. Spinal sensitiveness is an important aid in localisation, especially if it coincides with the sensory and motor phenomena. The most common and important spinal cord tumours are those of the meninges. Those growing within are nearly twice as common as those growing without the dura, the latter may exist for some time without giving rise to symptoms; the former cause symptoms early. The favourite site is the dorsal region, the lower and upper end. The histories of three cases are given at length. An important symptom in the first case was abdominal pain, which was diagnosed as appendicitis, and the author refers to other cases in which a similar diagnosis was made. In one the appendix was removed. In others gall-stones or indigestion was diagnosed. The author concludes his valuable paper with an abstract of 70 cases. Thirty of these were operated on, of which 12 were successful, eight partially so, and 10 wholly unsuccessful—that is to say, they died within a few weeks of the operation. Finally summing up, he says that 50 per cent. of intraspinal tumours are operable, and of these one-third to one-half are benefited by operation. When the surgeon can certainly exclude sepsis the results will be better. The earlier the case can be diagnosed the better is the result likely to be, and once tumour is diagnosed no time must be lost in giving drugs.

[6] Med. Rec., Dec. 6, 1902. [7] Med. Rec, Feb. 15, 1902.

